# Single‐Particle FRET Probes Heterogeneity in the Ligand Shell of Colloidal Perovskite Quantum Dots

**DOI:** 10.1002/adma.75051

**Published:** 2026-09-18

**Authors:** Leon G. Feld, Oleksandr Kolomiiets, Noah A. Shahin, Sebastian Sabisch, Amrutha Rajan, Gabriele Rainò, Maksym V. Kovalenko

**Affiliations:** ^1^ Laboratory of Inorganic Chemistry Department of Chemistry and Applied Biosciences ETH Zürich Zürich Switzerland; ^2^ Laboratory for Thin Films and Photovoltaics Empa – Swiss Federal Laboratories for Materials Science and Technology Dübendorf Switzerland

**Keywords:** colloidal quantum dots, Förster resonance energy transfer, ligands, perovskite, single‐molecule FRET, single‐particle spectroscopy

## Abstract

The functionality of colloidal quantum dots (QDs), whether in fundamental studies, optoelectronics, or emerging quantum technologies, critically depends on their surface chemistry, with organic ligands influencing nearly every aspect of their behavior. Whereas the inorganic core can be comprehensively characterized at atomic resolution, ligands remain difficult to probe owing to their low atomic contrast and soft, disordered structure. Here, we empower single‐particle photoluminescence (PL) spectroscopy to characterize the organic ligand shell. Tagging ligand tails with organic dyes enables measuring their distance from the QD via Förster resonance energy transfer (FRET) at sub‐nanometer resolution. Single‐particle, single‐molecule FRET experiments performed on lead halide perovskite QDs reveal nanometer‐scale variations in dye‐surface distances, both within individual particles (0.81 ± 0.22 nm on average) and between QDs with varied ligand chemistries (from 0.25 to 1.02 nm). Supported by atomistic modelling, our experiments tie diverse local ligand environments to these variations, validating a powerful yet accessible spectroscopic method for interrogating the complex surface chemistry of QDs, highly relevant to QD‐based optoelectronics, quantum applications, and photocatalysis.

## Graphical abstract

Single‐particle, single‐molecule Förster resonance energy transfer turns dye‐tagged ligands into nanorulers for the otherwise elusive organic shell of CsPbBr_3_ quantum dots. Distance variations within individual particles reveal pronounced local heterogeneity, while changes in the surrounding ligand chemistry shift the average dye–surface separation. Atomistic modeling supports diverse local ligand environments as a source of these variations.

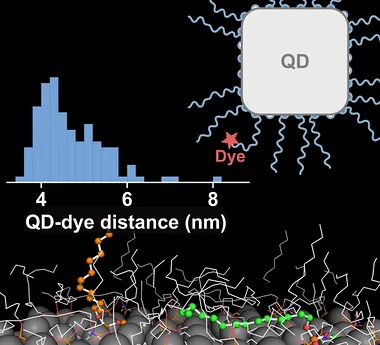

## 1 Introduction

Colloidal quantum dots (QDs)—solution‐synthesized semiconductor nanocrystals—are used in diverse applications, from light emission and detection to sensing, imaging, and catalysis. Their remarkable functionality and versatility stem from precisely tunable optical, electronic, and chemical properties. The entire lifespan and properties of QDs are influenced by surfactants that function as ligands (Figure 1a) [1]. For instance, ligands assist the QD formation [1, 2, 3, 4, 5], preserve semiconductor core integrity—essential for photostable (quantum) emitters [6, 7, 8]—and guide QD self‐assembly, which underpins fascinating collective optical and electronic phenomena [9, 10, 11, 12]. Moreover, ligands influence the interaction with reactants in photocatalysis [1, 13, 14]. They regulate coupling in QD films, enabling fast electronic transport in photodetectors (short ligands) or preserving high photoluminescence (PL) quantum yields (QYs) in down‐conversion films or light‐emitting diodes [15, 16, 17].

**FIGURE 1:**
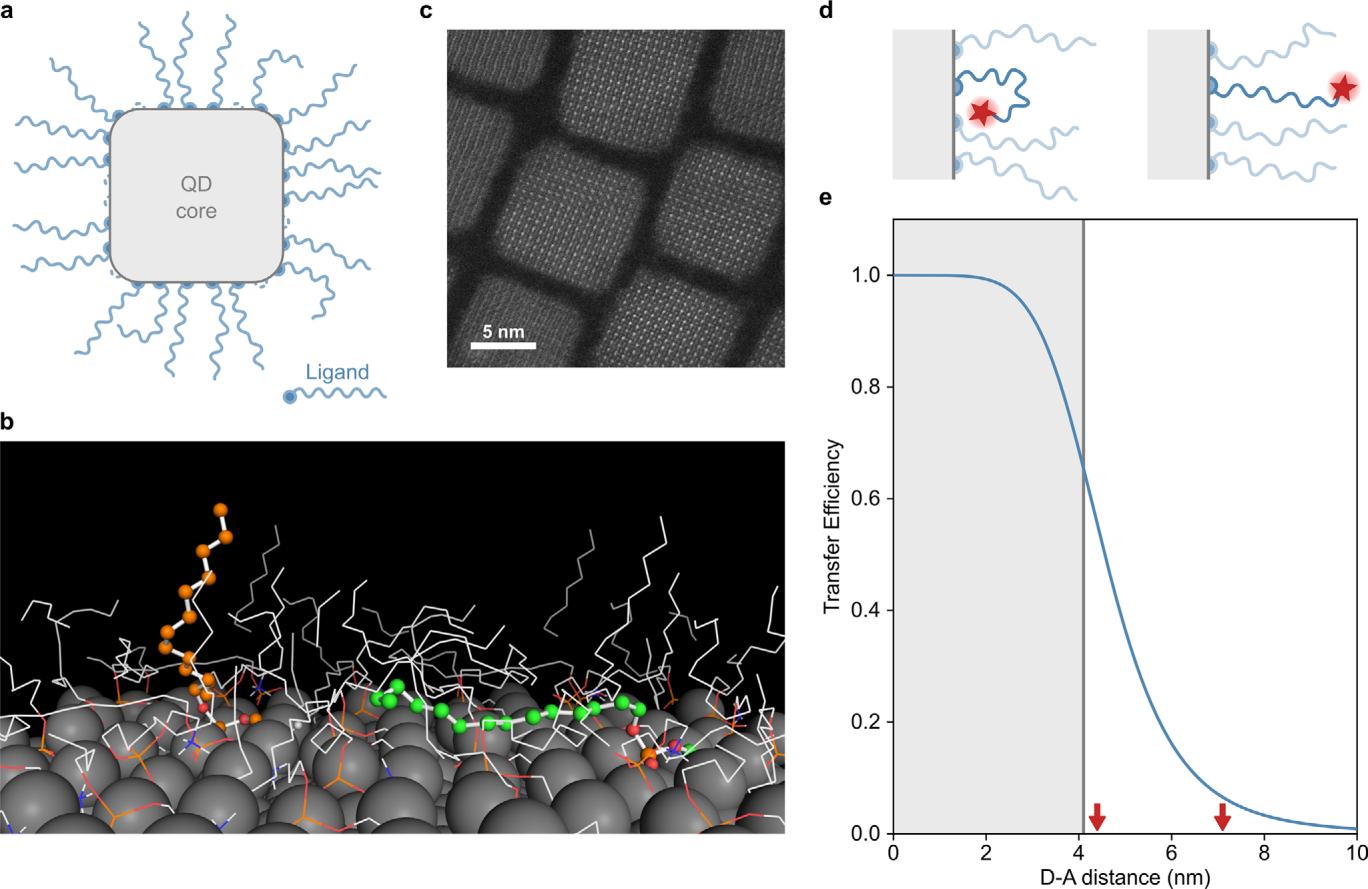
Concept of ligand mapping through FRET. (a) Schematic representation of a QD consisting of an inorganic semiconductor core and an organic ligand shell. (b) Snapshot of ligands on a CsPbBr_3_ surface from an MD simulation at 300 K. Two ligands are highlighted that represent the heterogeneity in ligand configurations and non‐polar hydrogens were omitted for clarity. (c) High resolution transmission electron microscopy image of CsPbBr_3_ QDs. (d) Scheme of a dye‐labelled ligand near the QD surface (left) and extending away from the surface (right). (e) Dependence of the FRET efficiency on the donor‐acceptor (D‐A) distance. The grey‐shaded area indicates the QD core and red arrows indicate the conformations illustrated in d.

Direct characterization of the configuration of organic ligands on the nanocrystal surfaces has remained challenging for decades. Vibrational and nuclear magnetic resonance (NMR) spectroscopy have been the primary methods to assess semiconductor‐ligand interaction [6, 18, 19]. They also offer insights into the conformation and packing of ligands; for instance, revealing the presence of gauche rotamers indicative of disorder in the ligand shell [20, 21, 22, 23]. Overall, NMR and vibrational spectroscopy are unmatched in probing chemical bonding in organic molecules but are inherently applicable only to large ensembles of QDs, failing to resolve heterogeneities across QDs and within the disordered ligand shell [1, 24]. A snapshot of the ligand shell, recorded during molecular dynamics (MD) simulations (details in Experimental Section), illustrates this disorder (Figure 1b). While transmission electron microscopy avoids ensemble‐averaging by offering atomic‐resolution imaging of single QDs, it does so only for their inorganic core [25], while the organic ligand molecules are largely invisible (Figure 1c). In this study, we turn to the relatively untapped potential of QD luminescence spectroscopy to obtain information on the organic ligand shell of single QDs.

Traditionally, steady‐state and time‐resolved photoluminescence (PL) spectroscopy evaluates the quality of QDs as emitters for their key applications, such as in television displays. When conducted at the single‐emitter level (single‐QD spectroscopy), one directly assesses the utility of QDs as quantum emitters for emerging and future applications in quantum information processing [26, 27, 28]. Furthermore, single‐QD spectroscopy inherently probes QD photophysics, unmasked from ensemble‐averaging effects, thereby aiding further structural and compositional engineering of QDs [28, 29, 30, 31]. PL properties are often rationalized by the inferred effects of organic ligand shells on the electronic “health” of the inorganic core of a QD, particularly the density of surface trap states, which govern, for instance, the PL quantum yield (QY), in relation to organic ligand density or the types of binding groups [6, 7, 8, 32, 33, 34]. At the same time, single‐particle PL spectroscopy of QDs has not been adapted to directly probe the organic ligand shell. Here, we implement single‐particle single‐molecule Förster resonance energy transfer (FRET) to probe the organic ligand shell at the molecular scale (Figure 1d). We showcase the method on organic‐ligand‐capped lead halide perovskite QDs [35]. Perovskite QDs have recently emerged as highly potent quantum emitters, owing to their ultrafast radiative rates and excitonic coherence [26, 36], and are, therefore, the subject of intense research with single‐QD PL methods [7, 27, 28, 37, 38, 39]. FRET proceeds via dipole‐dipole coupling with a distance dependence of R^−6^, providing a “spectroscopic ruler” [40, 41] (Figure 1e) to investigate ligand shell heterogeneity with nanoscale resolution. We designed a dye‐labelled ligand as a molecular probe to interrogate the organic ligand shell of CsPbBr_3_ perovskite QDs using a click‐chemistry approach, which is transferable to other colloidal QD platforms. Exploiting the method's high resolution, we observe and differentiate inter‐ and intra‐particle heterogeneities in the QD‐dye separation, studying a library of ligand tails. Our experimental single‐particle single‐molecule perspective, paired with atomistic insights from molecular dynamics (MD) simulations, propose variations in the local environment of the dye as the origin of the heterogeneity. The diversity of local ligand environments found here demonstrates the importance of single‐particle techniques to characterize the organic ligands of colloidal QDs.

## 2 Results and Discussion

### 2.1 Dye‐Labeled Ligands Through Click‐Chemistry

Our strategy to introduce dye‐labelled ligands as molecular probes for single‐particle single‐molecule FRET measurements into the organic ligand shell is summarized in Figure 2. When developing this strategy, we first sought to employ modular design principles to synthesize a molecular probe, adaptable to other colloidal QD materials. To this end, ligand **L1** features a phosphoethanolamine (PEA) group for surface coordination, an aliphatic chain as a spacer, and a strained alkyne as a tag (see Note S1 for the synthesis protocol). In contrast to traditional ligands of CsPbBr_3_ QDs, zwitterionic PEA‐based ligands exhibit slow ligand binding equilibria that are strongly tilted towards the surface‐bound state [13, 42]. For the adaptation to other colloidal QD materials, molecules resembling **L1** terminated by thiols, carboxylic acid, or amine head groups instead of PEA are commercially available.

**FIGURE 2:**
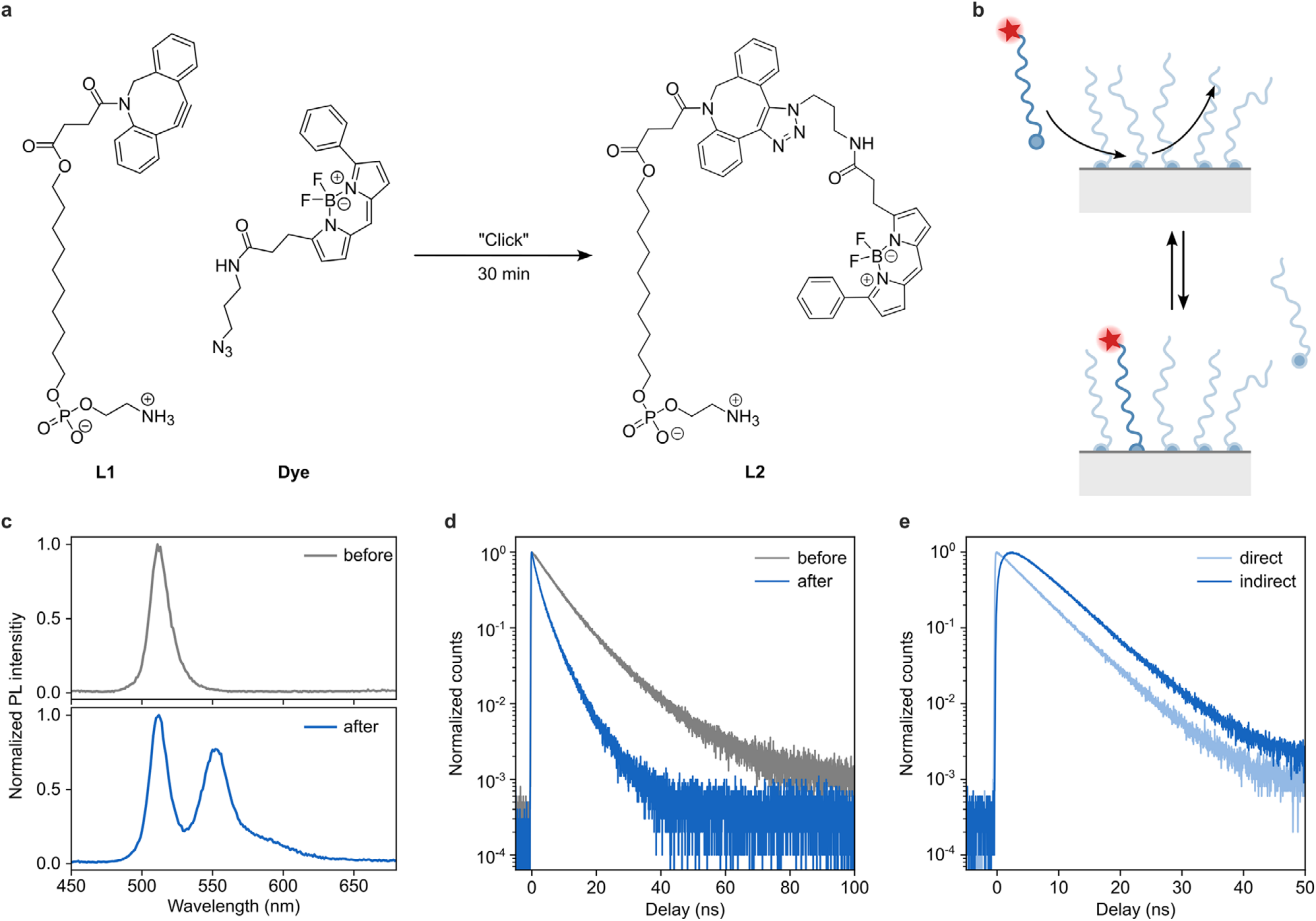
Synthesis of molecular probe and ligand exchange procedure. (a) Dye‐labelling of zwitterionic surface‐capping ligand **L1** through ring strain‐activated (catalyst‐free) click chemistry. (b) Schematic of the ligand‐exchange that introduces the molecular probe **L2** into the ligand shell. (c) Ensemble PL spectra of C_8_C_12_‐PEA capped CsPbBr_3_ QDs before and after ligand exchange. The samples were excited at 355 nm. (d) TRPL traces of the QD emission before and after ligand exchange upon excitation at 355 nm. (e) TRPL traces of the dye emission after ligand exchange upon direct excitation (532 nm excitation) and indirect excitation via FRET (355 nm excitation).

Termination with a dibenzocyclooctyne (DBCO) tag provides the flexibility to label the ligand with any azide‐containing dye via a facile and copper‐free click reaction. This is a key advantage considering that such dyes are commercially available and exist with absorption bands anywhere in the near‐UV to near‐IR range. We labelled **L1** with the charge‐neutral and bright (96% quantum yield) BODIPY‐derivative BDP R6G azide (*Lumiprobe*) to obtain the molecular FRET probe for this study (**L2**, Figure 2a). As shown in Figure S1, BDP R6G offers excellent spectral overlap between its absorption and the PL of CsPbBr_3_ QDs, a key requirement for efficient FRET.

We then focused on introducing **L2** via a procedure compatible with a wide range of original (native) ligands without further optimization. We therefore opted for a spontaneous entropically driven ligand exchange reaction replacing small amounts of native ligands by **L2** (Figure 2b). This approach exploits the reversible ligand binding observed for perovskite QDs, and we note that facile ligand exchange protocols are also available for other QD materials [43, 44, 45, 46]. Throughout this work, native ligands are based on the PEA binding group as their strong surface binding affords exceptionally high photostability and brightness in single QD PL experiments and the 2‐octyl‐1‐dodecyl‐PEA (C_8_C_12_‐PEA) variant showed high potential in (quantum) light emission applications [6, 7, 32]. We confirmed the completion of ligand exchanges and the occurrence of FRET through static and time‐resolved PL spectroscopy. Figure 2c–e shows such an analysis for CsPbBr_3_ QDs capped by C_8_C_12_‐PEA. Upon exchange, red dye emission (∼555 nm) emerges in addition to the QD emission (∼511 nm) in the PL spectrum upon excitation of QDs at 355 nm (Figure 2c). A shortened PL lifetime of the QDs after ligand exchange indicates the occurrence of FRET (Figure 2d). This is further supported by the rise time in the time‐resolved fluorescence trace of the dye emission, a hallmark of indirect excitation via FRET (Figure 2e).

### 2.2 Single‐Molecule FRET With Molecular Probe

For single‐particle single‐molecule FRET measurements, QDs treated with **L2** were diluted and spin‐coated into a polymer matrix to enhance the photostability of the emitters without altering the outcome of the experiments below (Figure S2). Measurements were performed in a custom‐built micro‐PL setup, employing pulsed excitation of QDs at 405 nm. The emission from single QD‐dye pairs is analyzed by acquiring PL spectra and performing time‐correlated single‐photon counting (TCSPC) in a Hanbury–Brown and Twiss experiment, modified to spectrally select dye‐ and QD‐emitted photons in its two detection arms, respectively [47]. To avoid QD‐QD energy transfer [48], spots featuring multiple QDs could be excluded by the inspection of the binary PL blinking of QDs (Figure S3) or based on the second‐order correlation measurements.

Figure 3a reports spectra of a single QD‐dye pair for a sample of CsPbBr_3_ QDs with C_8_C_12_‐PEA as native ligand. In the initial spectrum (blue), we observe emission by the QD donor and the dye acceptor. In a later spectrum (grey), QD emission has increased, while dye emission is absent. Consistent with that, the intensity traces of donor and acceptor recorded by TCSPC (Figure 3b) shows that photon counts from the acceptor abruptly decrease as the emission by the donor increases. This abrupt change is caused by photobleaching of the dye acceptor, and the number of such intensity drops signals the number of acceptor dyes [47].

**FIGURE 3:**
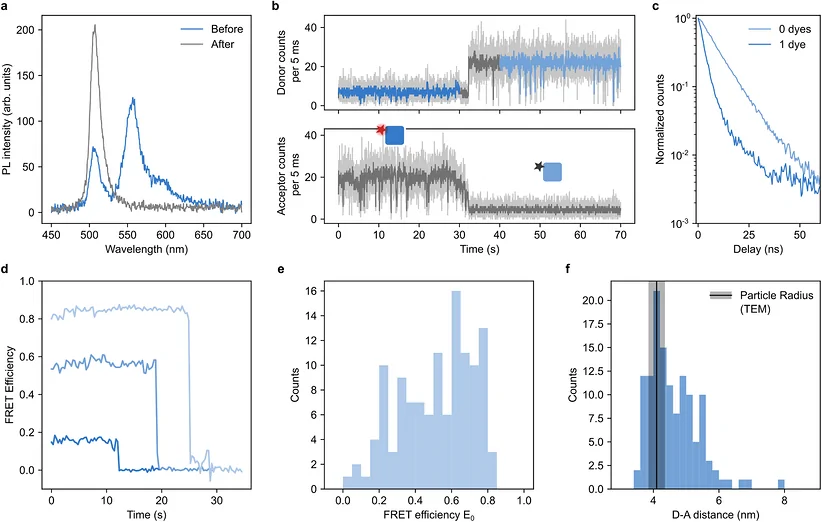
Single‐particle single‐molecule FRET for C_8_C_12_‐PEA capped CsPbBr_3_ QDs. (a) Representative PL spectra before (blue) and after (grey) photobleaching of acceptor dyes. (b) Temporal evolution of photon counts from a single QD donor (top) and a single dye acceptor (bottom). The step in intensity corresponds to photobleaching of the dye acceptor. Light grey traces represent the raw intensity traces to which a median filter (window size = 11) was applied to obtain the darker traces. Blue and light blue traces indicate sections for which TRPL traces displayed in c were constructed. (c) Single‐particle TRPL traces of the QD before (blue) and after photobleaching (light blue). (d) Traces of intensity‐based FRET efficiencies for individual QD‐dye pairs representing the heterogeneity. (e) Histogram of lifetime‐based FRET efficiencies (*E*_0_). (f) Histogram of the FRET‐derived donor‐acceptor (D‐A) distances. Black lines and areas indicate the particle radius and corresponding standard deviation determined by TEM.

Alongside observing dye fluorescence upon selective excitation of QDs, the anti‐correlation between donor and acceptor counts indicates the presence of FRET [47, 49, 50, 51]. Moreover, PL blinking in the donor emission is reproduced in the acceptor trace (Figure 3b). Finally, we can determine the time‐resolved PL of the donor before and after photobleaching of the dye. Figure 3c shows that photobleaching is accompanied by an increase in the donor lifetime, further corroborating the occurrence of FRET.

An estimation of the FRET efficiency, capable of delivering high temporal resolution, can be obtained from donor and acceptor intensities *I*_A_ and *I*_D_ as:

(1) $$E=\frac{I_{A}}{I_{A}+I_{D}}\;$$

Figure 3d reports the temporal evolution of the intensity‐based FRET efficiency for three separate QD‐dye pairs. The observation of FRET efficiencies ranging from around 15% to 85% suggests different donor‐acceptor distances (D‐A distances) which could correspond to different configurations (conformation, relative position, etc.) of **L2** on the QD surface.

For a systematic study of the FRET efficiency and D‐A distances, we estimate the FRET efficiency via the time‐resolved PL of the donor:

(2) $$E=1-\frac{\tau }{\tau_{0}}$$

where τ and τ_0_ are the donor lifetimes in the presence and absence of the acceptor, respectively. Lifetime‐based efficiencies are robust and require no correction of measured intensities [52]. We employ this estimation to extract *E*_0_, which is the FRET efficiency between a single donor and a single acceptor, for ∼200 QD‐dye pairs. The histogram of *E*_0_ shown in Figure 3e, confirms the initial suspicion of a pronounced heterogeneity across the ensemble of QD‐dye pairs.

Exploiting the (nano)ruler capability of FRET [40], the D‐A distance *R*_D − A_ can be estimated from *E*_0_ based on the expression,

(3) $$E_{0}=\frac{1}{1+{\left(\frac{R_{D-A}}{R_{0}}\right)}^{6}}\;$$

where *R*_0_ is the FRET radius, a constant that depends on several system‐specific parameters like spectral overlap of donor emission and acceptor absorption and the rotational averaging of donor and acceptor dipoles (further details in Note S2) [52]. Justified by the emission of CsPbBr_3_ QDs at room temperature being essentially unpolarized (Figure S4) and by (restricted) reorientation of the dye being allowed by the flexible linker, donor and acceptor dipoles are assumed to undergo dynamic isotropic averaging [53]. At small QD‐dye distances, Dexter‐type energy transfer, which also decays with increasing *R*_D − A_, may additionally contribute to measured *E*_0_. While the reduction of the QD by the dye could be energetically feasible [54, 55], there were no signs of charge transfer, like enhanced blinking or photodegradation [56], and an influence on *E*_0_ is thus unlikely.

Figure 3f shows the histogram of D‐A distances obtained from *E*_0_ values in Figure 3e. The sample‐averaged particle radius of 4.1(3) nm, determined by transmission electron microscopy (TEM, Figure S5), is shown for comparison. Figure 3f offers two key observations. First, the D‐A distances occupy a broad range (from 3.5 to 8 nm). This heterogeneity could be imposed by pronounced variations in the organic ligand shell that hosts the molecular probe **L2**. Second, a large fraction of **L2** adopts configurations with small D‐A distances and these are consistent with the TEM‐based particle radius, suggesting a tendency of the polar dye to approach the QD surface.

### 2.3 Intra‐Particle Variations Exceed Particle‐to‐Particle Variations

Next, we disentangle how particle‐to‐particle variations and variations within one particle contribute to the observed heterogeneity in measured D‐A distances. Therefore, we investigate FRET efficiencies for QDs loaded with multiple molecular probes. An example of donor and acceptor intensity traces for such a QD is shown in Figure 4a. In this case, three photobleaching steps result in the stepwise increase of donor counts and decrease of acceptor counts, confirming the presence of multiple (three) acceptor dyes.

**FIGURE 4:**
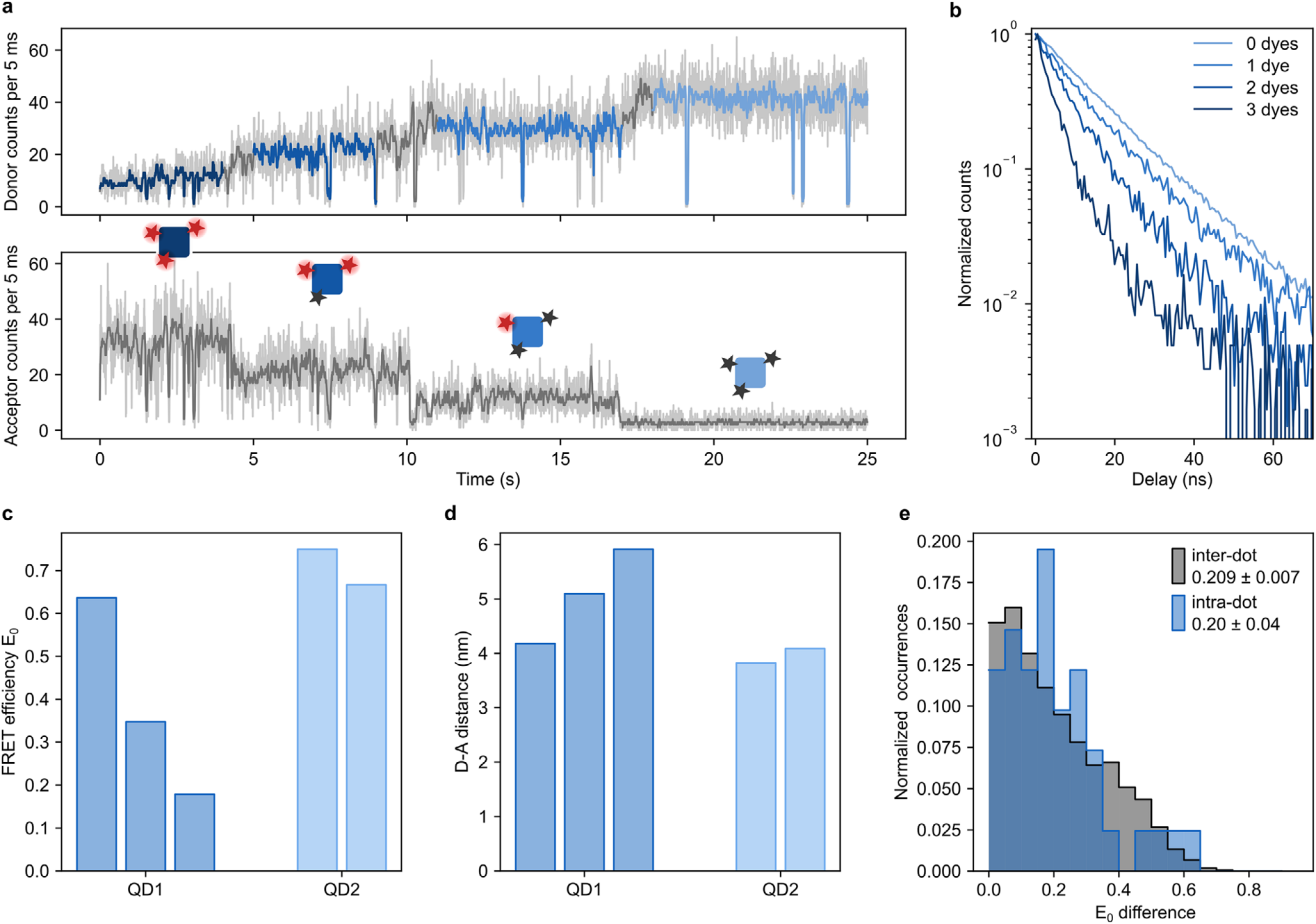
Intraparticle heterogeneity in single C_8_C_12_‐PEA capped CsPbBr_3_ QDs with multiple acceptors. (a) Temporal evolution of photon counts from a single QD donor (top) and multiple dye acceptors (bottom). Light grey traces represent the raw intensity traces to which a median filter (window size = 11) was applied to obtain the darker traces. Traces colored in different shades of blue indicate sections for which TRPL traces displayed in b were constructed. (b) Single‐particle TRPL traces of the QD for zero to three dye acceptors, colors encode the corresponding sections in a. (c) Lifetime‐based FRET efficiencies *E*_0_ for the dyes in (a) exhibiting large dye‐to‐dye variation and another QD (QD2) with a small dye‐to‐dye variation. (d) D‐A distances obtained from the *E*_0_ in (c). (e) Difference between *E*_0_ of different QDs (*inter*‐dot, grey) and between *E*_0_ recorded for the same QD (*intra*‐dot, blue).

All photobleaching events are accompanied by an increase in donor lifetime (Figure 4b) due to the total FRET efficiency scaling with dye‐loading [47, 48]. Obtaining the donor lifetime for each plateau enables the estimation of *E*_0_ for each acceptor (Note S3). Figure 4c compares the *E*_0_ extracted from Figure 4b (**QD1**) and another QD with two dye acceptors (**QD2**). They show very different behaviors. For **QD1**, all *E*_0_ differ greatly, ranging from 0.18 to 0.64, while the *E*_0_ for **QD2** are almost identical (both around 0.7). This translates into D‐A distances spanning a range of 1.7 nm for **QD1** and just 0.3 nm for **QD2** (Figure 4d). We can, therefore, rule out that all ligands coordinating to one QD exhibit identical D‐A distance, that is, that particle‐to‐particle variations are the only source of heterogeneity in *E*_0_. Moreover, a small bias towards smaller FRET efficiencies has been observed for QDs for which more than two *E*_0_ could be extracted, although this observation is based on a small sample size (Figure S6).

To unravel variations between and within particles, we dive into the statistics provided by ∼40 QDs with multiple acceptors and ∼60 QDs with a single acceptor. Our analysis shows that variations within individual QDs are dominant. To arrive at this conclusion, we calculated differences in *E*_0_ between dye acceptor molecules bound to the same QD donor (*intra*) and between acceptors bound to different donors (*inter*). Details of the analysis are described in Note S4. The histograms of *inter‐* and *intra‐*particle differences are shown in Figure 4e. There is no significant distinction between the two sets of differences, with a mean *intra*‐particle difference of 0.20 ± 0.04 and a mean *inter*‐particle difference of 0.209 ± 0.007. The analogous analysis of the D‐A distance reveals a mean *intra‐*particle difference of 0.81 ± 0.22 nm compared to a mean *inter‐*particle difference of 0.71 ± 0.03 nm. Therefore, we can conclude that variations within QDs fully account for the observed heterogeneity in *E*_0_. There are no significant effects that cause some QDs to exhibit systematically larger D‐A distances than other QDs. In other words, the molecular probe **L2** appears to encounter similar and, importantly, similarly diverse environments across all QDs.

### 2.4 Molecular Probe Responds to Host Ligands

To further test the reported method, we characterize how the environment, set by native ligands, influences the D‐A distance adopted by **L2**. This case study is enabled by the broad compatibility and straightforward extension of our labelling strategy to different native ligands, as well as the widely tunable ligand chemistry of colloidal QDs. We therefore prepare CsPbBr_3_ QDs with a range of PEA‐based ligands (Note S1), obtaining QDs with a library of native ligand tails including branched and linear aliphatic, aromatic, as well as more polar polyether tails (Figure 5a) [6]. After doping all organic ligand shells with **L2**, we performed complete ensemble PL and TRPL characterizations confirming ligand exchange and FRET (Figure S7).

**FIGURE 5:**
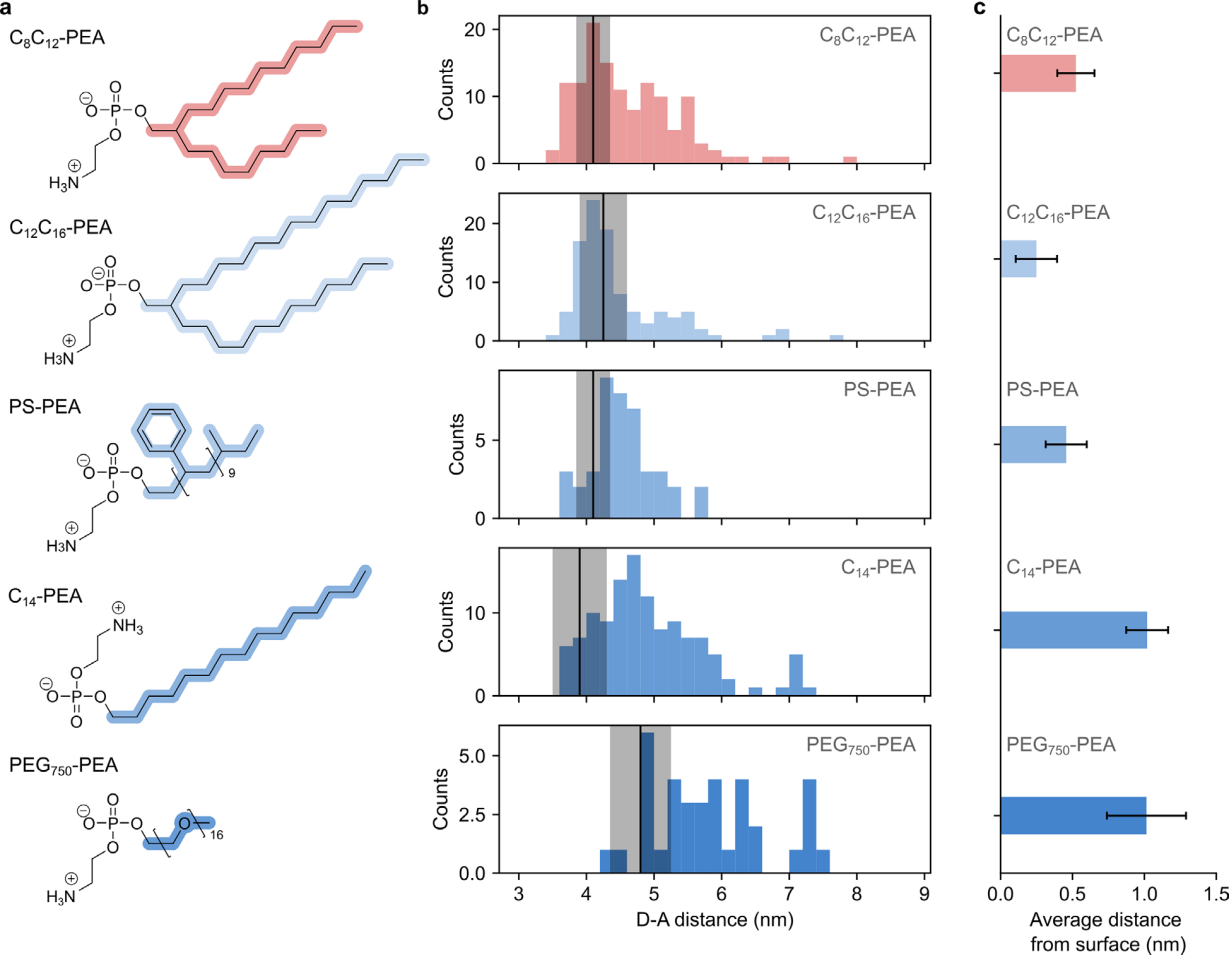
Single‐particle single‐molecule FRET for a library of ligand tails. (a) Structures of PEA‐based ligands with various tails used for single‐particle single‐molecule FRET studies. (b) Histograms of D‐A distances for ligands in (a). Black lines and areas indicate the particle radius and corresponding standard deviation determined by TEM. (c) Average distance between dye and QD surface (bar) and 95% confidence intervals (error bars).

This demonstration of the generalizability of the dye‐labelling strategy was followed by single‐particle single‐molecule FRET measurements. Histograms of the extracted D‐A distances alongside TEM‐based particle radii are reported in Figure 5b. As for C_8_C_12_‐PEA, we observe pronounced heterogeneities in D‐A distances across all types of native ligands. Moreover, we observe significant differences in the average distance of the acceptor from the QD surface (Figure 5c). Like the C_8_C_12_ tail, C_12_C_16_ and polystyrene (PS) favor small D‐A distances with acceptors residing near the QD surface. The apparent tendency to approach the perovskite surface can be explained by the dye's high polarity, and these tails appear unable to prevent this approach. On the other hand, C_14_ and PEG_750_ tails lead to significantly larger distances between the acceptor and QD surface. Compared to branched tails, linear aliphatic tails can exhibit a lower susceptibility to intercalation [57, 58], which could explain the larger average distance observed for C_14_. Meanwhile, being more polar, PEG_750_ could stabilize the polar dye farther from the QD surface. These observations demonstrate a profound influence of the environment on the D‐A separation adopted by individual molecular probes and confirm that our developed method captures this interplay among ligands, thereby demonstrating chemical control over ligand configuration.

### 2.5 Atomistic Insights Complement Experiment

Next, we aim to identify factors that cause the large heterogeneity in D‐A distances across dye‐QD pairs. We begin by pairing our experiments with atomistic insights through molecular dynamics (MD) simulations employing a density‐functional tight‐binding engine capable of handling large models. We construct models by placing **L2** on a CsBr‐terminated CsPbBr_3_ surface in the presence of 0% native ligands, C_14_‐PEA at 50% surface coverage, and PEG_750_‐PEA at 50% surface coverage, representing the upper limit of ligand loading previously quantified for C_8_C_12_‐PEA [59]. The resulting computational insights are reported in Figure 6. Representative snapshots of the structures of **L2** during MD simulations at 300 K are shown in Figure 6a–c. Additionally, Figure 6d–f report the potential energy as a function of the distance between the B‐atom in **L2** and the QD surface, alongside the corresponding average distance during the MD simulations. We observe that the average distances from MD simulations coincide with energy minima in scans of the potential energy surface (Figure 6d–f). Moreover, computational surface‐dye distances lie in a similar range (0.23 to 1.40 nm) as experimental ones (0.25 ± 0.14 nm to 1.02 ± 0.15 nm). These consistencies evidence the compatibility of the single‐particle single‐molecule experiments with computational insights.

**FIGURE 6:**
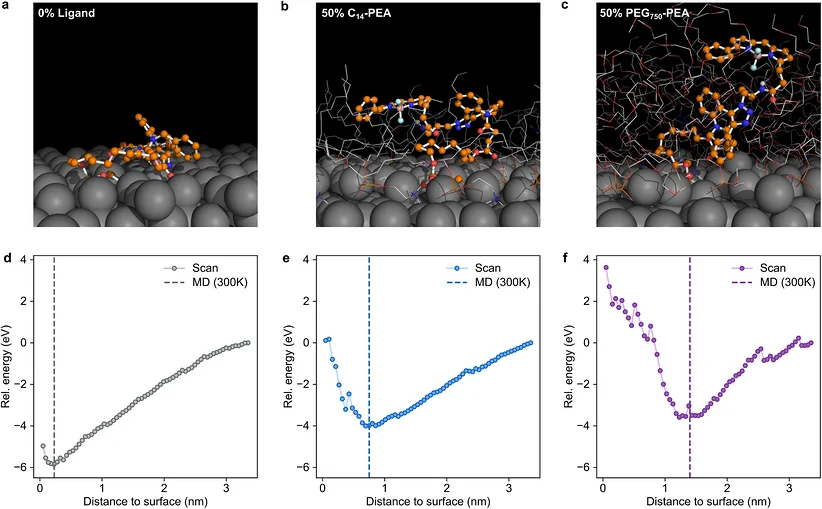
Atomistic modelling of dye‐labelled ligand in the ligand shell. (a) Representative snapshot of **L2** on the QD in absence of ligands from a 300 K MD simulation employing a density‐functional tight‐binding engine. (b) Representative snapshot of **L2** with 50% coverage with C_14_‐PEA ligands from a 300 K MD simulation employing a DFTB engine. (c) Representative snapshot of **L2** with 50% coverage with PEG_750_‐PEA ligands from a 300 K MD simulation employing a DFTB engine. (d–f) Potential energies of the systems in (a–c) as a function of the distance between the B‐atom of **L2** and the QD surface. Dashed lines indicate the average distance in 300 K MD simulations.

The observed increase in distance between 0% and 50% coverage confirms the experimental observation that the dye tends to reside near the surface (Figure 6a,d) and suggests that higher ligand coverage can reduce this tendency (Figure 6b,c,e,f). Furthermore, we observe that the composition of the native ligand tail affects the surface‐dye distance (Figure 6b,c,e,f). This generally agrees with experiments above (Figure 5c), although this difference was not observed experimentally between C_14_‐PEA and PEG_750_‐PEA. With these simulations in hand, we propose the local ligand coverage as the primary source of variations in the QD‐dye distance. This is consistent with the large heterogeneity observed within samples and within QDs, despite they exhibit identical native ligand tails. Local ligand coverage variations could be caused by ligands preferentially assembling in patches, previously postulated for several other colloidal QDs and nanoparticles [1, 60, 61], or facet‐dependent ligand binding strength to the three non‐equivalent facets of CsPbBr_3_ nanocubes [44, 61, 62]. The extent of the local ligand coverage variation could depend on the ligand tail and indeed different tails can feature different heterogeneities in the D‐A separation (Figure S8). Alternative sources of the observed heterogeneity include changes in the surface‐coordination facilitated by the reversible ligand binding of the PEA‐group or the relative position of the dye on the QD (facet, edge, corner, see Note S5) affecting the D‐A distance. Moreover, **L2** could be intrinsically disordered, that is, feature a diverse set of conformations which are adopted in the disordered ligand shell.

## 3 Conclusion

In summary, we have designed and demonstrated a micro‐PL‐based method that provides single‐molecule insights into the organic ligand shell of individual colloidal QDs. The method reveals nanometer‐scale variations in dye‐QD distances, suggesting diverse configurations adopted by the molecular probe within the ligand shell. Our experiments show that this inhomogeneity is caused by *intra*‐particle variations, while variations across different QDs from the same batch are negligible. Moreover, the composition of the native ligands that host the molecular probe influences the average dye‐QD distance, suggesting chemical control over the steric hindrance at the surface. These experiments point to the local environment as the differentiator of the behavior displayed by individual molecular probes and atomistic modelling proposes the local ligand coverage as a source of the observed heterogeneity. This locality is significant, for instance, for the interaction of QDs with degradants during optoelectronic operation or with reactants in photocatalysis. On the other hand, future studies may exploit single‐molecule insights from this tool to study the emergence of collective behavior of ligands that underpins macroscopic functionality. Finally, exploiting the nanosecond‐resolution of single‐molecule FRET [63] may ultimately offer dynamical insights into what this work captured only in a snapshot.

## 4 Experimental Section

### 4.1 Transmission Electron Microscopy

Transmission electron microscopy (TEM) images were collected by using a JEOL Model JEM‐2200FS microscope operated at 200 kV.

HR HAADF‐STEM was performed using a probe‐aberration‐corrected FEI Titan Themis system, operated at 300 kV. High‐resolution images were obtained by summing up 3−5 frames.

### 4.2 NMR Measurements

Spectra for routine characterization of products of organic syntheses were measured on a Bruker Avance III HD Widebore NMR or a Bruker Avance III HD nano bay spectrometer operating at a proton frequency of 400 MHz and equipped with a 5 mm probe. Chemical shifts are referenced to the CDCl_3_ deuterium signal and reported in parts per million (ppm). Coupling constants (J) are reported in Hz, and a peak's multiplicity is specified with the following notation: s = singlet, d = doublet, t = triplet, m = multiplet, bs = broad singlet.

### 4.3 Ligand Exchange Protocol

In a nitrogen‐filled glovebox, washed colloidal QD solutions (200 µL, 1.8–3 mg/mL) were combined with **L2** (20–40 µL, 0.1 mM) and diluted in dichloromethane (ACROS, 99.8% extra dry over molecular sieves, filtered) to a total volume of 300 uL. To ensure equilibration in the ligand exchange, samples were left at least overnight prior to measurements.

### 4.4 Single‐Particle FRET Measurements

*Sample preparation*. After ligand exchange, QD samples with C_8_C_12_‐PEA, C_12_C_16_‐PEA, and C_14_‐PEA were diluted in two steps by a factor of 10 000 in *n*‐octane (Acros, 99+% extra dry) and once by factor 10 in a 1 mass‐% solution of Polystyrene‐*block*‐poly(ethylene‐*ran*‐butylene)‐*block*‐polystyrene (SEBS; Aldrich, average M_w_ ≈ 118’000) in cyclohexane (99.5% extra dry over molecular sieve, filtered). QD samples with PS‐PEA and PEG_750_‐PEA were diluted in two steps by a factor of 10 000 in toluene (Acros 99.85% extra dry over molecular sieve, filtered) and by a factor of 2–20 in a 3 mass‐% solution of polystyrene (Aldrich, average *M*_w_ ≈ 280 000) in toluene (Acros 99.85% extra dry over molecular sieve, filtered). At 150 revolutions per second for 60 s, 150 µL of diluted samples were spin‐coated onto clean precision cover glasses (Thorlabs, 170 ± 5 µm thickness, Ø25 mm diameter). Films were placed in a custom‐built air‐tight sample cell. All steps were prepared in a nitrogen‐filled glovebox.

*Measurements* were performed in a custom‐built micro‐PL setup that resembles an epifluorescence microscope. Single‐QD films mounted on XYZ translational stages (SmarAct, <1 nm resolution) were irradiated by a 405 nm pulsed excitation laser (PicoQuant, 1–10 MHz repetition rate, <50 ps pulse width, <100 W/cm^2^) that was focused using an oil immersion objective (1/e^2^ = 1 µm, 1.3 NA). Light collected through the same objective was passed through a 90/10 beamsplitter, excitation was rejected by a long‐pass filter (450 nm cut‐on wavelength) and analyzed in modified HBT setup or in a monochromator coupled to an EMCCD (Princeton Instruments, one frame per second). The modified HBT setup consisted of two avalanche detectors (Excelitas, 250 ps time resolution), a single‐photon counting module (PicoQuant, HydraHarp), a 50/50 beamsplitter, a long‐pass filter in one arm (550 nm cut‐on wavelength), and a bandpass filter (510 nm central wavelength, 40 nm bandwidth) in the second arm. Measurements were performed by focusing on a single‐QD spot and acquisition of PL spectra for several seconds before and after single‐photon counting in the HBT setup for 60–120 s. Single QDs were identified based on their binary blinking. Intensity traces from Figures 3 and 4 are displayed alongside background traces in Figure S12.

*Data analysis* was performed in custom python scripts. Donor decays were constructed for periods between photobleaching steps, and the decay was fitted by a bi‐exponential decay without offset and the 1/e decay time was determined. Detailed information on the lifetime‐derived FRET efficiencies and the differentiation of *inter*/*intra*‐particle variations are reported in Notes S3 and S4, respectively.

### 4.5 Time‐Resolved Photoluminescence

PL spectra and TRPL traces were acquired under ambient conditions in a commercial PL spectrometer (Picoquant, FluoTime 300) upon exciting with a 355 nm pulsed laser (Picoquant, 2.5 MHz repetition rate). Donor and acceptor decays were measured at the peak center of the QD emission and 555 nm, using a 1.5 nm bandwidth.

### 4.6 Atomistic Modelling

We used a published room temperature crystal structure of CsPbBr_3_ [64] to prepare a xy‐periodic slab corresponding to a 3‐by‐3‐by‐1 array of lead‐centered perovskite unit cells, creating a CsBr‐terminated surface. Based on previous insights [6], ligands were placed on the surface by replacing a Cs‐Br unit by the ammonium & phosphate coordinating groups. For 50% ligand coverage, every second row of Cs‐Br was fully displaced by ligand, that is, two of the four potential nearest neighboring sites around each ligand are occupied. Images of such a slab are provided in Figure S13.

*Simulations* were performed in ADF 2024.102 [65], using the GFN1‐xTB model [66]. Molecular dynamics (MD) simulations in the NVT‐ensemble at 300 K (1 fs timestep, ≥ 40 000 frames) employed a Berendsen thermostat (10 fs damping constant). To constrain ion diffusion and retain the crystal structure, Pb and Br atoms were frozen and bonds within the ammonium were constrained by a harmonic potential. The average distance of the B‐atom of **L2** from the slab surface was extracted and averaged over the last 10 ps, before which total energy and dye‐surface distances have fully equilibrated.

For potential energy surface scans of the distance of the B‐atom of **L2** from the slab surface, ligand shells were first equilibrated in MD simulations, followed by scanning the z‐coordinate of the B‐atom in steps of ∼0.5 Å and geometry optimization at each distance. To avoid artefacts from these relatively large and abrupt steps, an additional constraint was imposed on the bonds of the B‐atom using a harmonic potential.

### 4.7 Statistical Analysis

*Error representation*. Standard deviations are represented in brackets, and 95% confidence intervals are provided using “±”.

## Author Contributions

**Leon G. Feld**: conceptualization, investigation, writing – original draft, methodology, validation, visualization, writing – review and editing, formal analysis, data curation. **Oleksandr Kolomiiets**: conceptualization, investigation, writing – review and editing, visualization, methodology, data curation. **Noah A. Shahin**: investigation, writing – review and editing, data curation, visualization, conceptualization. **Sebastian Sabisch**: investigation, writing – review and editing, data curation, conceptualization. **Amrutha Rajan**: investigation, resources, data curation. **Gabriele Rainò**: conceptualization, writing – original draft, methodology, writing – review and editing, project administration, supervision. **Maksym V. Kovalenko**: conceptualization, writing – review and editing, funding acquisition, project administration, supervision, resources.

## Funding

This publication was created as part of NCCR Catalysis (Grant Nos. 180544 and 225147), a National Centre of Competence in Research funded by the Swiss National Science Foundation (SNSF). O.K. acknowledges financial support from the SNSF through “Ukrainian‐Swiss Joint Research Projects, 2023‐UA‐CH‐NANO” (Grant No. IZURZ2_224907). A.R. acknowledges financial support from ETH Grants (LOOP, ETH‐32.22‐1).

## Conflicts of Interest

The authors declare no conflicts of interest.

## Supporting Information

## Supporting information




**Supporting File**: adma75051‐sup‐0001‐SuppMat.docx.
Supporting Notes 1–6, Table S1, and Figures S1–S13.

## Data Availability

All data supporting the findings in this study is available through Zenodo (https://doi.org/10.5281/zenodo.18229476).
