# Supplementary material for: Single‐Particle FRET Probes Heterogeneity in the Ligand Shell of Colloidal Perovskite Quantum Dots
Source: Adv Mater. 2026 Sep 18;38(62):e75051. doi: 10.1002/adma.75051 (PMC13741126; doi:10.1002/adma.75051)
Supplement: Supplementary file 1 [file ADMA-38-e75051-s001.docx]

**Supplementary Information for**

**Single-particle FRET Probes Heterogeneity in the Ligand Shell of Colloidal Perovskite Quantum Dots**

Leon G. Feld,^1,2^ Oleksandr Kolomiiets,^1,2^ Noah A. Shahin,^1,2^ Sebastian Sabisch,^1,2^ Amrutha Rajan,^1,2^ Gabriele Rainò,^1,2,*^ Maksym V. Kovalenko^1,2,*^

^1^ Laboratory of Inorganic Chemistry, Department of Chemistry and Applied Biosciences, ETH Zürich, CH-8093 Zürich, Switzerland

^2^ Laboratory for Thin Films and Photovoltaics, Empa – Swiss Federal Laboratories for Materials Science and Technology, CH-8600 Dübendorf, Switzerland

*Corresponding author. Email: [mvkovalenko@ethz.ch](mailto:mvkovalenko@ethz.ch), [rainog@ethz.ch](mailto:rainog@ethz.ch)

**Contents**

Supplementary Note 1. Ligand and QD synthesis	p.2

Supplementary Note 2. Förster Radii	p.6

Supplementary Table 1	p.6

Supplementary Note 3. Determination of E_0_ for donors with multiple acceptors	p.7

Supplementary Note 4. Comparison of inter- and intra-particle variations of E_0_	p.7

Supplementary Note 5. Influence of the particle shape	p.7

Supplementary Note 6. NMR spectroscopy of click reaction	p.8

Supplementary Figures	p.9
**Supplementary Note 1.** Ligand and QD synthesis

*Materials.* All chemicals for organic synthesis were used as supplied without further purification. Dry solvents were purified with a solvent purification system. *Reagents for ligand synthesis*: phosphorus(V) oxychloride (99%, Sigma-Aldrich); triethylamine (99%, Sigma-Aldrich); 2-aminoethan-1-ol (>=99.0%, Sigma-Aldrich); acetic acid (>99.8%, Sigma-Aldrich). *Alcohol substrates for ligands synthesis*: 1-Tetradecanol (99+%, Acros); 2-Octyl-1-dodecanol (97%, Sigma-Aldrich); 2-Dodecylhexadecan-1-ol (>93%, TCI); ω-Hydroxyterminated polystyrene (Mn=900 P4465-SOH, Polymer Source, Inc.); Poly(ethylene glycol) monomethyl ether (Mr=750 ABCR, Aldrich). *Substrates for synthesis of DBCO-C_10_-OH*: 11,12-Didehydrodibenzo[*b,f*]azocin-5(6*H*)-yl)-4-oxobutanoic acid *(*DBCO-acid, 98%, Ambeed); 4-Dimethylaminopyridine (>99.0%, TCI); *N*,*N*′-Dicyclohexylcarbodiimide (99%, Sigma-Aldrich). *Reagents for NC synthesis*: Lead (II) bromide (PbBr2, 99.999%), cesium carbonate (Cs2CO3, 99.9%) were purchased from Sigma-Aldrich. Diisooctylphosphinic acid (DOPA, 90%) were purchased from Fluorochem. *n*-Octane (min. 99%) was purchased from Carl Roth. Trioctylphosphine oxide (TOPO) min. 90% was purchased from Strem Chemicals. Acetone (extra dry, 99.8%), hexane (≥99%), ethyl acetate (EtOAc), toluene (anhydrous, 99.8%) and cyclohexane (CH) were purchased from Sigma-Aldrich.

1.1 Synthesis of native ligands

The ligand syntheses were adapted from our previous publication; see Ref. [1].

*Aliphatic chains.^[1]^* A solution of the alcohol substrate (0.025 mol; 1 equiv.) and triethylamine (0.0275 mol; 1.1 equiv.; 3.83 mL) in 25 ml of dry tetrahydrofuran (THF) was added dropwise to a solution of POCl_3_ (0.03 mol, 1.2 eq., 2.78 mL) in dry THF (2.5 mL) which was vigorously stirred and kept over an ice water bath. After addition, the reaction mixture was stirred at room temperature for 20 min. The reaction mixture was then placed in a room temperature water bath, followed by the dropwise addition of ethanolamine (0.03 mol; 1.2 equiv.; 1.81 mL) and triethylamine (0.06 mol; 2.4 equiv.) in THF (37.5 mL) under vigorous stirring. After addition, the reaction temperature was increased to 40°C for 20 min. The reaction mixture was filtered, and the filtrate solution was dried. The oily residue was dissolved in *n*-hexane (20 mL) and stirred until a precipitate formed. The precipitate was filtered out, and the filtrate was evaporated to yield alkyl-2-oxo-1,2,3-oxazaphospholane. The compound was dissolved in a mixture of acetic acid (5.7 mL) and distilled water (2.6 mL) at 70°C for 35 minutes, then the product was precipitated by addition of acetone (125–150 mL). The precipitate obtained was washed three times with cold acetone and then dried overnight under a vacuum at 40–50°C.

The ^1^H and ^31^P NMR spectra of C_8_C_12_-PEA, C_12_C_16_-PEA, and C_14_-PEA were in full agreement with previously reported data.^[1]^

*Polystyrene chain.^[1]^* A solution of the alcohol substrate (0.025 mol; 1 equiv.) and triethylamine (0.0275 mol; 1.1 equiv.; 3.83 mL) in 25 mL of dry tetrahydrofuran (THF) was added dropwise to a solution of POCl_3_ (0.03 mol, 1.2 eq., 2.78 mL) in dry THF (2.5 mL) which was vigorously stirred and kept over an ice water bath. After addition, the reaction mixture was stirred at room temperature for 20 min. The reaction mixture was then placed in a room temperature water bath, followed by the dropwise addition of ethanolamine (0.03 mol; 1.2 equiv.; 1.81 mL) and triethylamine (0.06 mol; 2.4 equiv.) in THF (37.5 mL) under vigorous stirring. After addition, the reaction temperature was increased to 40°C for 20 min. The reaction mixture was filtered, and the filtrate solution was dried. The oily residue was dissolved in a mixture of acetic acid (5.7 mL), distilled water (2.6 mL), and THF (2 mL) at 70°C for 35 minutes, then the product was precipitated by addition of water (125–150 mL). The precipitate obtained was washed three times with water and then dried overnight under a vacuum at 40–50°C.

The ^1^H and ^31^P NMR spectra of PS-PEA were in full agreement with previously reported data.^[1]^

*Polyethylene glycol (PEG) chain.^[1]^* Monomethyl ether of poly(ethylene glycol) (average molecular mass 750, MeOPEG750, 0.025 mol, 1 equiv.) and triethylamine (0.0275 mol, 1.1 equiv., 3.83 mL) in 25 mL of dry THF were added dropwise to a solution of POCl_3_ (0.03 mol, 1.2 eq.) in dry THF (2.5 mL) kept over an ice water bath. After addition, the reaction mixture was stirred at room temperature for 15 min. The reaction mixture was then placed in a room temperature water bath, followed by the dropwise addition of ethanolamine (0.03 mol; 1.2 equiv.; 1.81 mL) and triethylamine (0.06 mol; 2.4 equiv.) in THF (37.5 mL) under vigorous stirring. After addition, the reaction temperature was increased to 40°C for 15 min. The reaction mixture was filtered, and the filtrate solution was dried. The oily residue was dissolved in a mixture of acetic acid (5.7 mL) and distilled water (2.6 mL) at 70°C for 30 minutes. The product was separated from the reaction mixture as a viscous oil by addition of acetone (10 eq. by volume) and *n*-hexane (10-20 eq. by volume). To the oily residue a small amount of chloroform was added, followed by addition of excess diethyl ether to precipitate the ligand. The washing was repeated several times. The product was dissolved in chloroform, kept over activated molecular sieves for 30 min. The solution was filtered through a 0.22 μm PTFE filter, chloroform was evaporated, and the obtained viscous liquid was placed under Schlenk line vacuum at 30-40°C overnight.

The ^1^H and ^31^P NMR spectra of PEG-PEA were in full agreement with previously reported data.^[1]^

1.2 Synthesis of **L1**

*DBCO-C_10_-OH.* 4-(11,12-Didehydrodibenzo[*b,f*]azocin-5(6*H*)-yl)-4-oxobutanoic acid *(*DBCO-acid) (2.5 g, 8.18 mmol) and decane-1,10-diol (4.28 g, 24.56 mmol) were dissolved in anhydrous CH_2_Cl_2_ (40 mL) and THF (20 mL) under an argon atmosphere. To the stirred solution at 25 °C, a solution of DCC (1.70 g, 8.20 mmol) in CH_2_Cl_2_ (40 mL) and DMAP (200 mg, 1.64 mmol, catalytic amount) was added. The reaction mixture was stirred at 25°C for 24 h, and the resulting precipitated DCU was removed by filtration. The combined filtrate was concentrated under reduced pressure, diluted with CH_2_Cl_2_ (10 mL), and filtered again to remove residual solids. The filtrate was then washed twice with a saturated NaHCO_3_ solution, followed by a brine solution. It was dried over Na_2_SO_4_ and concentrated under reduced pressure to afford a yellow oil. The crude product was purified by column chromatography on silica gel (SiO_2_ - 300 mL; eluent: CH_2_Cl_2_/MeOH = 60:1, second spot on TLC: target compound, R_f_ = 0.3). The resulting viscous yellowish liquid was dried under vacuum overnight to give the product (1.25 g).

Yield 33%. ^1^H NMR (400 MHz, CDCl_3_) δ 7.70 (dd, J = 7.6, 1.3 Hz, 1H), 7.57 – 7.48 (m, 1H), 7.46 – 7.24 (m, 6H), 5.18 (d, J = 13.8 Hz, 1H), 4.07 – 3.91 (m, 2H), 3.75 – 3.59 (m, 3H), 2.69 (dddd, J = 46.1, 17.2, 8.2, 6.4 Hz, 2H), 2.34 (dt, J = 17.2, 6.2 Hz, 1H), 1.97 (dt, J = 16.4, 6.1 Hz, 1H), 1.56 (m,5H), 1.41 – 1.22 (m, 12H); ^13^C NMR (101 MHz, CDCl_3_) δ 172.98, 171.72, 151.49, 148.04, 132.27, 129.32, 128.51, 128.27, 128.10, 127.72, 127.08, 125.47, 125.44, 123.15, 122.68, 114.94, 107.73, 64.69, 63.04, 55.49, 32.8, 29.48, 29.46, 29.39, 29.37, 29.26, 29.16, 28.47, 25.82, 25.72.

***L1***. A solution of *DBCO-C_10_-OH* (1.25 g, 2.7 mmol; 1 equiv.) and triethylamine (2.97 mmol; 1.1 equiv.; 414 µL) in 6 ml of dry THF (6 mL) was added dropwise to a solution of phosphorus oxychloride (3.24 mmol; 1.2 equiv.; 303 µL) in 1 mL THF which was vigorously stirred and kept on a cooling bath. After addition, the reaction mixture was stirred at room temperature for 40 min. The reaction mixture was then placed in an ice bath, followed by the dropwise addition of ethanolamine (3.24 mmol; 1.2 equiv.; 196 µL) and triethylamine (6.48 mmol; 2.4 equiv.; 903.3 µL) in THF (6 mL) under vigorous stirring. After addition, the reaction was stirred at room temperature before heating to 40°C for 40 min. The reaction mixture was placed in a freezer and centrifuged to remove precipitate. The solution was evaporated, and the residue was dissolved in a mixture of acetic acid (616 μL) and distilled water (281 μL) at 70°C for 35 min. The product was separated by precipitation in acetone (washed in acetone with consequent 3 times). The product was dissolved in DCM (8 mL), filtered through a 0.22 μm PTFE filter, and the solvent was evaporated to yield a yellowish product. Then 30ml of hexane was added to precipitate **L1**, followed by solidifying **L1**, and then decanting the liquid phase. The solid was dried overnight and stored in a freezer inside a glovebox.

Yield 56%. ^1^H NMR (400 MHz, CDCl_3_) δ 8.54 (bs, 3H), 7.70 – 7.63 (m, 1H), 7.53 – 7.46 (m, 1H), 7.42 – 7.19 (m, 6H), 4.08 (s, 2H), 4.02 – 3.87 (m, 2H), 3.80 (q, *J* = 6.7 Hz, 2H), 3.65 (d, *J* = 13.8 Hz, 1H), 3.16 (s, 2H), 2.66 (dddd, *J* = 48.6, 17.2, 8.3, 6.3 Hz, 2H), 2.30 (dt, *J* = 17.2, 6.1 Hz, 1H), 1.92 (dt, *J* = 16.4, 6.1 Hz, 1H), 1.54 (m, 4H), 1.25 (m, 12H); ^13^C NMR (101 MHz, CDCl_3_) δ 172.94, 171.68, 151.50, 148.05, 132.26, 129.33, 128.51, 128.25, 128.09, 127.72, 127.07, 125.46, 123.16, 122.66, 114.93, 107.76, 64.66, 55.47, 31.59, 29.61, 29.58, 29.45, 29.29, 29.22, 28.53, 25.90, 25.82, 22.66, 14.13; ^31^P NMR (162 MHz, CDCl_3_) δ 0.02.

1.3 Click Reaction to **L2.**

*Preparation of* ***L1*** *stock solutio*n. **L1** ligand (39.4 mg, 0.0675 mmol) was dissolved in CDCl_3_ (0.675 mL) to afford a 0.1 M solution. The solution was mixed until it was completely dissolved and used directly for subsequent reactions.

*Preparation of* ***BDP R6G azide*** *stock solution.* **BDP R6G azide** (5.0 mg, 0.0118 mmol) was dissolved in dry dichloromethane (1.0 mL) to obtain a 0.0118 M solution. The solution was stirred or vortexed until it was fully dissolved, and then it was used without further purification.

*Click reaction.* An aliquot of the **BDP R6G azide** solution (150 µL, 1.77 µmol, 0.0118 M) was combined with **L1** solution (17.7 µL, 1.77 µmol, 0.10 M) in a vial. The reaction mixture was gently mixed and allowed to react at room temperature for 30 min. The reaction progress and product formation were assessed by ^1^H, ^1^H-^1^H and ^1^H-^13^C NMR spectroscopy, see Supplementary Note 6 and Supplementary Fig. 9-11.

1.4 Quantum dot synthesis

The QD synthesis and purification were adapted from Ref. [1].

*Stock Solutions*.^[1]^ A PbBr_2_-TOPO stock solution was prepared from PbBr_2_ (1 mmol, 367 mg) and TOPO (90%) (5.5 mmol, 2144 mg) dissolved in *n*-octane (5 mL) at 120 °C on a hot plate under ambient conditions. Pb precursor was then diluted with hexane (20 mL). A Cs-DOPA stock solution was obtained by reacting Cs_2_CO_3_ (100 mg) with DOPA (1 mL) in octane (2 mL) at 120°C, followed by dilution with hexane (27 mL). Ligand stock solution (0.1 M) was prepared by dissolving 0.1 mol of ligand in 1 mL of CHCl_3_.

*Synthesis and Purification*.^[1]^ A 4 mL glass vial was loaded with PbBr_2_-TOPO stock solution (500 µL, 0.04 M) and hexane (750 µL). Upon vigorous stirring, Cs-DOPA stock solution (250 µL, 0.02 M) was injected, and the solution was left stirring for 90 s. The solution was then treated with a ligand stock solution (60 μL of ligand, 0.1 M in CHCl_3_) and stirred for 90 s. The crude solution was then destabilized with 2 volume equivalents of acetone (3 mL) or hexane (3 mL, in the case of the Polystyrene-PEA ligand), followed by centrifugation at 20133 g for 1 min, and subsequently redispersed in 1 mL of a good solvent (cyclohexane, toluene, or CHCl_3_). The purification step was then repeated one more time to obtain colloids with native ligands.

**Supplementary Note 2. Förster Radii**

The Förster radius R_0_ for a donor-acceptor pair is defined as

$R_{0}=0.221 \left( \frac{\kappa^{2} \Phi_{D}J\left( \lambda\right)}{n^{4}} \right)^{\frac{1}{6}}$,

with the orientation factor $\kappa^{2}$, the donor photoluminescence quantum yield $\Phi_{D}$ (we used 0.95), the spectral overlap of donor emission and acceptor absorption $J\left( \lambda\right)$, and the refractive index $n$. We assumed $\kappa^{2}$ = 2/3, which corresponds to fast randomization of donor and acceptor dipoles. Considering that FRET distances scale only with the sixth root of $\kappa^{2}$, that the QD emission is unpolarized, and that the acceptor is bound via a flexible linker, this assumption remains reasonable even if the acceptor dipole is not completely randomized. We roughly estimate the refractive index of the medium as

$n=\frac{2}{3} 2.4+\frac{1}{3} 1.37=2.06$ ,

where we chose 2.4 as the refractive index of the QD core and 1.37 (refractive index of hexane) for the organic ligands and polymer.

The spectral overlap integrals $J\left( \lambda\right)$ were obtained using *a|e - UV-Vis-IR Spectral Software 2.2* (FluorTools, [www.fluortools.com](http://www.fluortools.com)). For perovskite QDs, small differences between the PL spectrum in the ensemble and at the single-particle level may occur. Thus, we determined the average PL peak center of single QDs. Then, we used a PL spectrum of a CsPbBr_3_ QD ensemble with a similar peak center and the absorption spectrum of BDP R6G azide to determine $J\left( \lambda\right)$. We note that – within a sample – correlations between single-particle single-molecule FRET efficiencies and PL peak center are not apparent, given the large heterogeneity. Below, Supplementary Table 1 reports the obtained R_0_ values.

**Supplementary Table 1.** Average single-particle PL center for different native ligands and the PL center of a reference ensemble spectrum used to calculate the FRET radius R_0_.

| Native ligand | Average PL center, Single-particle PL (nm) | PL center, reference spectrum (nm) | R_0_ (nm) |
| --- | --- | --- | --- |
| C_8_C_12_-PEA | 507(3) | 507 | 4.588 |
| C_12_C_16_-PEA | 507(4) | 507 | 4.588 |
| PS-PEA | 508.2(1.0) | 507 | 4.588 |
| C_14_-PEA | 504(7) | 502 | 4.466 |
| PEG_750_-PEA | 500.0(2.2) | 502 | 4.466 |

**Supplementary Note 3. Determination of E_0_ for donors with multiple acceptors**

The lifetime-based FRET efficiency E_0_ for a single D-A pair is

$E_{0}^{(1)}=1-\frac{\tau_{1}}{\tau_{0}}$	,

Where $\tau_{1}$ and $\tau_{0}$ are the donor lifetimes before and after photobleaching. Similarly, if there is another step, we can obtain E_0_ for the other D-A pair

$E_{0}^{(2)}=1-\frac{\tau_{2}}{\tau_{1}}$	,

$\tau_{2}$ being the lifetime in presence of both dyes.

Because periods between steps can be short, we chose to determine the FRET efficiency $E_{n}= 1-\frac{\tau_{n}}{\tau_{0}}$ for each step and reconstruct all $E_{0}^{(n)}$ using information from the entire trace. We obtained

$E_{0}^{(2)}=\frac{\frac{E_{n=2}}{1-E_{n=2}} - \frac{E_{0}^{(1)}}{1-E_{0}^{(1)}}}{1+\frac{E_{n=2}}{1-E_{n=2}} - \frac{E_{0}^{(1)}}{1-E_{0}^{(1)}}}$	and	$E_{0}^{(3)}=\frac{\frac{E_{n=3}}{1-E_{n=3}} - \frac{E_{0}^{\left( 2 \right)}}{1-E_{0}^{\left( 2 \right)}}- \frac{E_{0}^{\left( 1 \right)}}{1-E_{0}^{\left( 1 \right)}}}{1+\frac{E_{n=3}}{1-E_{n=3}} - \frac{E_{0}^{\left( 2 \right)}}{1-E_{0}^{\left( 2 \right)}}- \frac{E_{0}^{\left( 1 \right)}}{1-E_{0}^{\left( 1 \right)}}}$	.

**Supplementary Note 4. Comparison of *inter*- and *intra*-particle variations of E_0_**

The number of acceptors per QD is small, mostly being two or three. Therefore, common metrics to measure variations, like variance, standard deviations, or mean absolute errors, are not available to assess the intra-particle variations. Thus, we chose to determine the absolute pair-wise difference in E_0_ between each dye of the same QD

$D_{ij}=\left| E_{0}^{\left( i \right)}- E_{0}^{\left( j \right)} \right|$	.

The same analysis is performed to assess inter-particle variations by computing the pairwise differences between dyes across different QDs. Normalized histograms of the two sets of pairwise differences are plotted in Figure 4e.

**Supplementary Note 5. Influence of the particle shape**

CsPbBr_3_ QDs samples exhibit cuboidal shapes with varying degrees of truncation at the corners (Supplementary Fig. 5). This could introduce a broadening of the measured D-A distance histograms (heterogeneity), even at uniform dye-surface separation. Amongst the studied samples, particle shapes for QDs capped by PS-PEA, PEG_750_-PEA, and C_12_C_16_-PEA appear more spherical than the other samples, discernible by the profile and packing in TEM images (Supplementary Fig. 5). Whereas PS-PEA capped QDs exhibit narrower D-A distance histograms (large D-A distances are not observed), PEG_750_-PEA and C_12_C_16_-PEA lead to similar heterogeneity as less truncated QD samples (Supplementary Fig. 8). That the particle shape largely accounts for the observed heterogeneity is therefore unlikely.

**Supplementary Note 6. NMR spectroscopy of the click reaction.**

All spectra were recorded on a Bruker Avance III HD Spectrometer equipped with a 5 mm Prodigy CryoProbe operating at a proton frequency of 500.13 MHz (11.7 T). Measurements were performed at 298 K and referenced to the CDCl_3_ deuterium signal. To track the reaction the characteristic ^1^H signals at 5.2 and 3.7 ppm of two neighboring protons in the eight-membered ring were monitored (Supplementary Fig. 9a). These signals shift to 6.2 and 4.4 ppm upon addition of the dye as the click reaction induces a conformation change of the ring (Supplementary Fig. 10a). Due to the complexity of the dye-ligand complex and the fact that both protons show scalar coupling to each other, ^1^H-^1^H COSY spectra were acquired before and after the click reaction (Supplementary Fig. 9b, Supplementary Fig. 10b). The assignment of these signals is further supported by ^1^H-^13^C HSQC spectra, indicating that the two protons are bound to the same carbon (Supplementary Fig. 9c, Supplementary Fig. 10c). After the reaction the spectrum did not contain any residual peaks of unreacted DBCO suggesting full conversion of the ligand.

1D ^1^H spectra were acquired using a 90° excitation pulse and a recycle delay of 15 s. The T^1^ relaxation times of the dye, ligand and complex were determined using inversion recovery experiments with the slowest relaxing proton signal showing a relaxation time of 3 s. ^1^H-^1^H COSY spectra were acquired using a phase-sensitive pulse sequence featuring multiple quantum selection and gradients for coherence selection. For each spectrum, two scans and 256 transients were recorded. ^1^H-^13^C HSQC spectra were recorded using a phase sensitive pulse sequence using gradient for coherence selection featuring shaped pulses, multiplicity editing and decoupling during the acquisition. The delays were set to select for a scalar coupling of 145 Hz. For each spectrum four scans and 256 transients were recorded.

**Supplementary Figures**

**Supplementary Fig. 1.** Ensemble photoluminescence (PL) of C_8_C_12_-PEA capped CsPbBr_3_ QDs and absorbance (abs.) of BDP R6G azide (*Lumiprobe*).

**Supplementary Fig. 2.** Influence of the polymer (SEBS) matrix on the observed FRET distances for C_8_C_12_-capped CsPbBr_3_ QDs. **a,** D-A distances measured for QDs embedded in a SEBS film (Fig. 3f). **b**, D-A distances measured for bare QDs on glass (no SEBS). The mean D-A distances are displayed in the upper right corner of each panel with their 95% confidence interval.

**Supplementary Fig. 3.** Donor (top) and acceptor (bottom) intensity time traces illustrating features used to reject spots containing two or more QDs. Blue and red traces were obtained by applying a median filter (width = 11 points) to the raw data (lighter traces). Grey shaded area: QD1 without an acceptor is emitting, while QD2 is almost fully quenched by an active dye. Purple arrows indicate some of the instances where QD1 turns OFF due to blinking. Yellow shaded area: QD1 is emitting and QD2, for which the dye is now photobleached, is turned OFF due to blinking. White areas: QD1 and QD2 are both emitting. Yellow arrows indicate some of the instances where QD2 turns OFF (like the yellow shaded area).

**Supplementary Fig. 4.** Polarization-dependent PL measurements of two single C_8_C_12_-PEA capped CsPbBr_3_ QDs. Top: PL spectra as a function of linear polarizer angle in the detection path. Bottom: Integrated PL spectra as a function of linear polarizer angle in the detection path.

**Supplementary Fig. 5.** TEM images and measured edge lengths for CsPbBr_3_ QD samples.

**Supplementary Fig. 6.** Dependence of E_0_ on the number of extractable E_0_ per QD for C_8_C_12_-PEA capped CsPbBr_3_ QDs. **a-c,** Histograms for the observed E_0_ for QDs with one (a), two (b), and three (c) extractable E_0_. The grey histogram represents all E_0_, n denotes the number of measured QDs. **d,** Mean E_0_ as a function of the number of extractable E_0_. Error bars indicate 95% confidence intervals, and the black line and shaded area correspond to the mean and 95% confidence interval across all spots.

**Supplementary Fig. 7.** Ensemble PL and TRPL characterization for further samples. **a,** Ligands used in this study, excl. C_8_C_12_-PEA. **b-e,** Ensemble PL spectra (355 nm excitation) for samples with the ligands in a before and after ligand exchange with **L2**.**f-i,** TRPL traces of the donor emission for the ligands in a before and after ligand exchange with **L2** (355 nm excitation).**j-m,** TRPL traces of the acceptor emission for the ligands in a after ligand exchange with **L2** upon indirect (355 nm) excitation via FRET and direct excitation (532 nm).

**Supplementary Fig. 8.** Variation in the FRET distance for different native ligands.

**Supplementary Fig. 9.** ^1^H (a), ^1^H-^1^H COSY (b) and ^1^H-^13^C HSQC spectra of **L1.**

**Supplementary Fig. 10.** ^1^H (a), ^1^H-^1^H COSY (b) and ^1^H-^13^C HSQC spectra of **L2.**

**Supplementary Fig. 11.** ^1^H (a), ^1^H-^1^H COSY (b) and ^1^H-^13^C HSQC spectra of BDP R6G azide (**dye**)**.**

**Supplementary Fig. 12.** Intensity time traces and background signal. **a,** Donor (top) and acceptor (bottom) traces for a C_8_C_12_-PEA capped CsPbBr_3_ QDs featuring one acceptor, including a background trace (green). The dark grey traces were obtained by applying a median filter (width = 11 points) to the raw photon counts (light grey). **b,** Donor (top) and acceptor (bottom) traces for a C_8_C_12_-PEA capped CsPbBr_3_ QDs featuring three acceptors, including a background trace (green). The dark grey traces were obtained by applying a median filter (width = 11 points) to the raw photon counts (light grey).

**Supplementary Fig. 13.** Atomistic model of **L2** in the presence of C_14_-PEA at 50% ligand coverage.

**References**

(1)	V. Morad, et al. Designer phospholipid capping ligands for soft metal halide nanocrystals. *Nature* **2024**, *626* (7999), 542–548, DOI: 10.1038/s41586-023-06932-6
